# Interleukin-17 Contributes to Chikungunya Virus-Induced Disease

**DOI:** 10.1128/mbio.00289-22

**Published:** 2022-03-07

**Authors:** Xiang Liu, Yee-Suan Poo, Juliana C. Alves, Roque P. Almeida, Helen Mostafavi, Patrick Chun Hean Tang, Richard Bucala, Mauro M. Teixeira, Adam Taylor, Ali Zaid, Suresh Mahalingam

**Affiliations:** a Menzies Health Institute Queensland, Griffith University, Southport, Queensland, Australia; b School of Medical Sciences, Griffith University, Southport, Queensland, Australia; c Global Virus Network Centre of Excellence in Arboviruses, Griffith University, Southport, Queensland, Australia; d Division of Immunology and Molecular Biology Laboratory, University Hospital/EBSERH, Federal University of Sergipe, Aracaju, Sergipe, Brazil; e Department of Internal Medicine, Yale University School of Medicine, New Haven, Connecticut, USA; f Department of Biochemistry and Immunology, Universidade Federal de Minas Gerais, Belo Horizonte, Brazil; Johns Hopkins Bloomberg School of Public Health

**Keywords:** alphavirus, chikungunya, interleukin-17

## Abstract

Alphaviral arthritides caused by mosquito-borne arboviruses such as chikungunya virus (CHIKV) can persist for months after the initial acute disease. Here, we investigated the contribution of interleukin-17 (IL-17), a cytokine involved in chronic autoimmune arthropathies such as rheumatoid arthritis, to the development of alphaviral arthropathy. Sera from CHIKV-infected patients who displayed both acute and chronic disease showed high levels of IL-17, IL-6, IL-21, IL-22, and IL-23, especially during the chronic phase of disease. We sought to validate these findings using a mouse model of CHIKV infection and disease using wild-type and IL-17A-deficient mice. Mice were infected with CHIKV, and joint and muscle tissues were harvested at designated time points. Tissue infiltrates were examined by immunohistochemistry, and tissue mRNA and protein expression of cytokines was assessed. Joint and muscle pathology was assessed using histology. CHIKV-infected mice lacking IL-17A showed reduced tissue inflammation and neutrophil infiltration, compared to wild-type mice. These investigations showed a role for IL-17 in the acute phase of CHIKV infection and also during the postacute disease resolution phase.

## INTRODUCTION

Mosquito-borne viruses, or arboviruses, are a group of pathogens of major public health concern in light of several recent outbreaks. Reemerging arboviruses comprise two major families of RNA viruses, i.e., flaviviruses (e.g., Zika virus [ZIKV]) and alphaviruses (e.g., chikungunya virus [CHIKV], Ross River virus [RRV], and Mayaro virus [MAYV]), each with specific geographic distribution, mosquito vector specificity, and disease etiology (1, 2). Arthritogenic alphaviruses, including CHIKV, RRV, MAYV, and o’nyong’nyong virus (ONNV), are primarily associated with rheumatic disease, which is characterized by acute fever and rash, and classically acute and chronic polyarthritis/polyarthralgia, which can be debilitating and protracted (1, 2). Up to 40% of patients experience persistent joint inflammation and arthralgia, which can persist for months or years after infection (3, 4). These viruses are a major cause of infectious arthropathies worldwide, and recent outbreaks of CHIKV have helped highlight the need for intervention strategies (1, 3). While CHIKV disease (CHIKVD) can be treated with nonsteroidal anti-inflammatory drugs and/or paracetamol, treatment often provides inadequate relief (3). Inflammatory cytokines such as interleukin 6 (IL-6), IL-1β, and tumor necrosis factor (TNF) have been shown to be biomarkers of both acute and chronic CHIKVD (5), with levels of these cytokines also commonly being elevated in autoimmune arthritides such as rheumatoid arthritis (RA). A cytokine implicated in RA pathogenesis, IL-17, is often associated with chronic inflammatory conditions (6) and is also the target of monoclonal antibody (MAb) immunotherapy to treat inflammatory (including musculoskeletal) conditions (7). IL-17 is expressed by CD4^+^ and CD8^+^ T cells, as well as γδ T cells and subsets of innate lymphoid cells (ILCs) (6). While the role of T-cell-derived IL-17A in alphavirus infection is not known, studies have shown that CD4^+^ T cells are important effectors of CHIKV arthritis (8, 9), and blocking their infiltration into infected tissues in mice resulted in reduced pathology (10). CD4^+^ T cells can differentiate into IL-17-producing T cells under certain inflammatory conditions, particularly when IL-6 and IL-23 are present (6). In addition to T-cell-derived IL-17, several reports over the years have shown that neutrophils, a subset of innate immune cells that form part of most inflammatory responses, including arthritis, can also produce IL-17, especially in the context of an IL-6-rich environment (11). In this study, we assessed the presence of IL-17A and IL-17-pathway-related proteins in patients with acute and chronic CHIKVD and, using an experimental mouse model of CHIKVD, we investigated the role of IL-17A in pathogenesis. This study, which is based on clinical data and an animal model, indicates a role for IL-17 during the acute and postacute phases of CHIKV infection.

## RESULTS

### IL-17A is associated with acute and chronic CHIKVD in patients.

**FIG 1:**
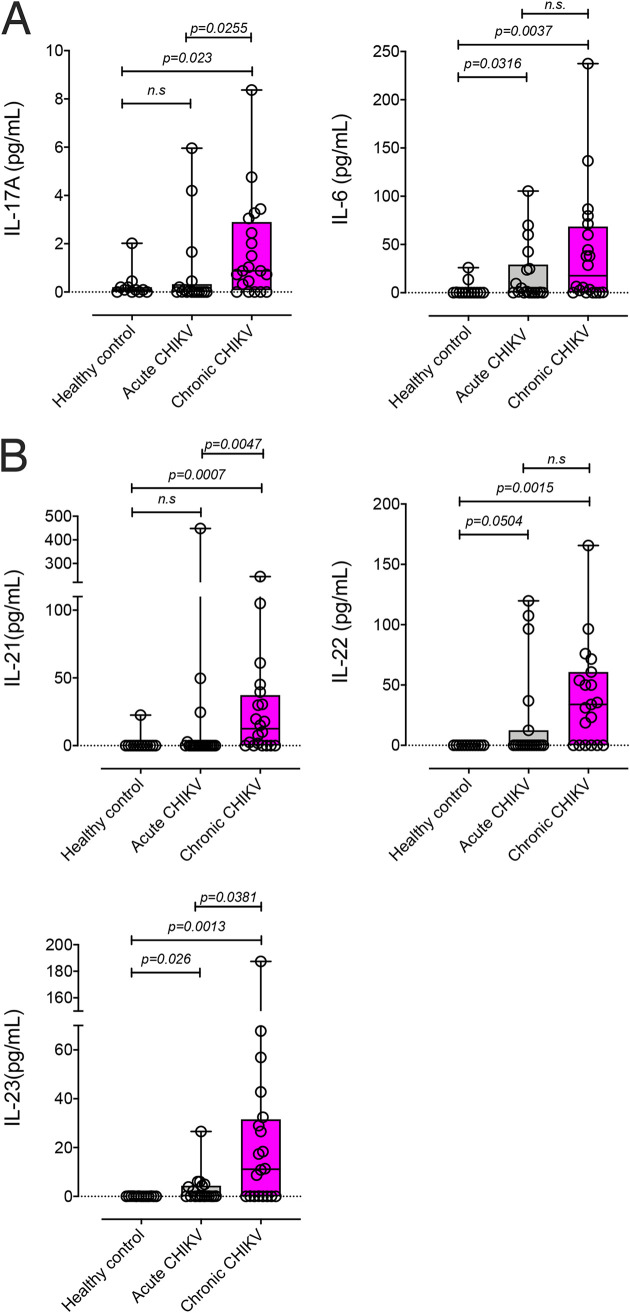
CHIKV patient cytokine measurements. IL-17A and IL-6 (A); IL-21, IL-22, and IL-23 (B) levels were measured in patients with acute CHIKV (∼4 days postonset) or chronic CHIKV (∼143 days postonset). Each data point represents an individual patient, and cytokine levels are shown in picograms per milliliter of serum. Statistical significance of cytokine levels in acute CHIKV patients versus healthy controls and in chronic CHIKV patients versus healthy controls was assessed by a one-way ANOVA. *P* values are shown on the plots.

Alphavirus-induced pathophysiology (severe arthritis and myositis) and RA share several features (9). IL-17–producing immune cells, particularly T cells, have been implicated in the immunopathology of RA, and we asked whether IL-17 represented a reliable correlate of CHIKV-induced arthritis in infected patients. Serum samples from CHIKV-infected patients (Table 1) were collected during the 2018 outbreak in northern Brazil and were assessed for the presence of IL-17A, IL-6, IL-21, IL-22, and IL-23 by enzyme-linked immunosorbent assay (ELISA). Patients were screened for other arboviral infections such as ZIKV and dengue virus (DENV) and were assessed for typical clinical manifestations associated with CHIKV, including fever, arthralgia, myalgia, and rash. CHIKV patients who were on average 4 days after disease onset were categorized as acute cases, and those who were on average 166 days postonset were categorized as chronic cases (Table 1). Some patients with acute CHIKV showed elevated IL-17A levels in the serum, but chronic CHIKV patients showed greater and significant increases in IL-17A levels, compared to healthy controls (Fig. 1A). Similarly, higher levels of IL-6 were measured in three acute CHIKV patients, but the majority of chronic CHIKV subjects showed significantly higher levels of IL-6, compared to healthy controls (Fig. 1A). We also assessed serum levels of the IL-17-pathway-associated cytokines IL-21, IL-22, and IL-23. Similar to our observations with IL-17A and IL-6, while a small number of acute CHIKV patients showed elevated levels of IL-21, IL-22, and IL-23, these cytokines were most elevated in sera of chronic CHIKV patients (Fig. 1B). Of note, in contrast to chronic CHIKV patients, the majority of cytokine readouts were found to be below the detection limit for acute CHIKV patients (Fig. 1A and B).

**TABLE 1:** CHIKV patient information summary^*a*^

Parameter	Data for:		
	Healthy controls (*n* = 13)	Acute CHIKV patients (*n* = 20)	Chronic CHIKV patients (*n* = 20)
CHIKV positive (% [no. positive/total no.])			
CHIKV PCR positive	Untested	100 (20/20)	100 (20/20)
CHIKV IgG positive	0 (0/12)	Untested^*b*^	41.1 (7/17)
CHIKV IgM positive	0 (0/3)	Untested	72.2 (13/18)
Age (mean ± SEM) (yr)	39.5 ± 2.86	34.0 ± 2.99	49.58 ± 2.69
Time from disease onset to sample collection (mean ± SEM) (days)	0	4.31 ± 1.59	166 ± 18.6
Signs/symptoms (% [no. positive/total no.])			
Fever	0	95 (19/20)	90 (18/20)
Arthralgia	0	95 (19/20)	100 (20/20)
Rash	0	65 (13/20)	80 (16/20)
Myalgia	0	80 (16/20)	95 (19/20)
Medication for symptoms (% [no. positive/total no.])	0 (0/13)	90 (18/20)	95 (19/20)
Anti-inflammatory	0 (0/13)	35 (7/20)	70 (14/20)
Antipyretic	0 (0/13)	70 (14/20)	60 (12/20)
Analgesic	0 (0/13)	50 (10/20)	40 (8/20)
DENV/ZIKV positive (% [no. positive/total no.])			
DENV IgG positive	0 (0/8)	Untested	Untested
ZIKV IgG positive	8.3 (1/12)	Untested	Untested
ZIKV PCR positive	Untested	0 (0/20)	0 (0/20)

a A total of 53 adult subjects (including 13 healthy controls) were recruited for this study in the state of Sergipe (Brazil) following admission to the University Hospital in Aracaju. Serology and PCR screens were used to confirm CHIKV cases and exclude other endemic arboviral (DENV and ZIKV) infections. Acute CHIKV patients presented to the hospital an average of 4.31 days after the onset of symptoms, while chronic CHIKV patients presented an average of 166 days postonset. A summary of prescribed medications specific for CHIKVD symptoms is shown (see Table S1 in the supplemental material for additional details).

b Group in which only 1 of 20 patients underwent a CHIKV IgG test.

10.1128/mbio.00289-22.1 TABLE S1 Supplemental table. Download Table S1, XLSX file, 0.02 MB. Copyright © 2022 Liu et al. 2022 Liu et al. https://creativecommons.org/licenses/by/4.0/ This content is distributed under the terms of the Creative Commons Attribution 4.0 International license.

### IL-17A deficiency ameliorates acute CHIKVD in mice.

**FIG 2:**
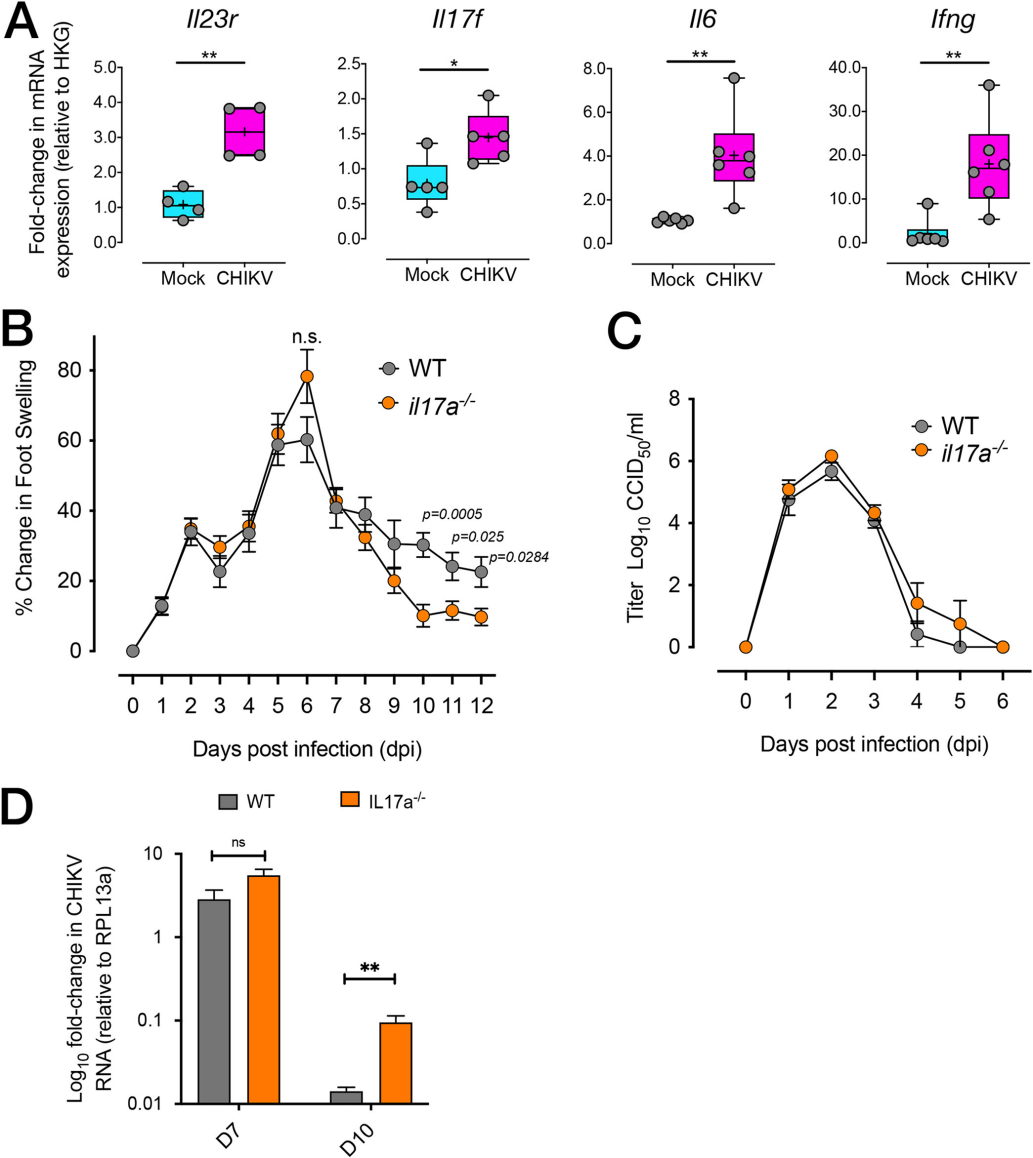
CHIKV-induced joint inflammation is reduced in the absence of IL-17. (A) mRNA expression of *Il23r*, *Il17f*, *Il6*, and *Ifng* in the joint tissue of mock-infected or CHIKV-infected C57BL/6J mice at 10 dpi. mRNA expression is expressed as fold change relative to gene expression in mock-infected mice and is normalized to housekeeping gene expression. *, *P* < 0.05; **, *P* < 0.01, Mann-Whitney *U* test. (B) Foot swelling (expressed as change in foot height times width, compared to day 0) in WT (C57BL/6J) and *il-17a^−/−^* mice infected with CHIKV. Statistically significant differences between groups at each time point were assessed using a Kolmogorov-Smirnoff test. ns, not significant. (C) CHIKV viremia in WT and *il17a^−/−^* mice infected with CHIKV. Viral titers are expressed as log_10_ CCID_50_ per milliliter of serum. (D) Relative CHIKV RNA levels determined by qRT-PCR in the feet of WT (C57BL/6J) mice (*n* = 7 per time point) and *il-17a^−/−^* mice (*n* = 4 per time point) harvested 7 and 10 days after infection. CHIKV RNA levels were normalized to those of *Rpl13a* (housekeeping gene). Differences on day 7 were not significant. **, Differences on day 10 were significant, Kolmogorov-Smirnov test, *P = *0.012.

To determine whether IL-17 has a role in CHIKV infection, an adult mouse model of infection and disease was used (12). Gene expression analysis of the feet of CHIKV-infected wild-type (WT) mice revealed significant upregulation of *Il23r*, *Il17f*, *Il6*, and *Ifng* mRNA expression at 10 days postinfection (dpi), compared to uninfected mice (Fig. 2A). To determine whether IL-17A deficiency would lead to reduced pathology in CHIKV-infected mice, WT and *il17a^−/−^* knockout mice were infected subcutaneously in the feet with CHIKV and monitored for foot swelling for 12 days. While levels of early foot swelling were similar in *il17a^−/−^* mice and WT mice, WT mice displayed more prolonged foot swelling than did *il17a^−/−^* mice, with *il17a^−/−^* mice showing significantly reduced swelling on 10, 11, and 12 dpi (Fig. 2B). A lack of IL-17A was not associated with significant differences in CHIKV viremia between *il17a^−/−^* and WT mice (Fig. 2C). However, quantitative real-time Reverse Transcription (qRT)-PCR for CHIKV RNA showed that, while amounts of viral RNA in the feet were not significantly different at 7 dpi, feet from *il17a^−/−^* mice contained significantly more CHIKV RNA than did those from WT mice on 10 dpi (Fig. 2D).

### Reduced inflammation in IL-17A-deficient CHIKV-infected mice.

**FIG 3:**
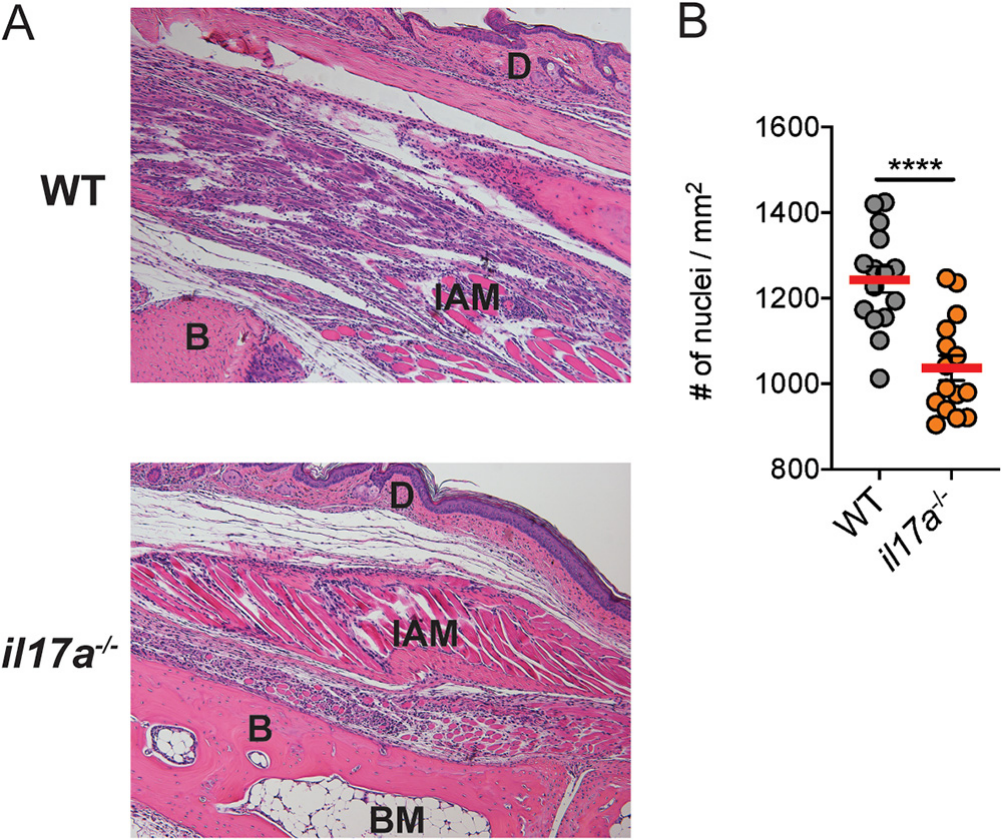
CHIKV-induced joint inflammation is reduced in the absence of IL-17. (A) H&E staining of WT and *il17a^−/−^* mouse feet following infection with CHIKV at 10 dpi. BM, bone marrow; B, bone; D, dermis. (B) Quantification of nuclear stain in nonconsecutive sections of H&E-stained foot sections from WT and *il17a^−/−^* mice at 10 dpi. Statistical analysis of differences between groups was performed using a Mann-Whitney *U* test. ****, P < 0.001. Data are representative of 2 independent experiments (*n* = 6 mice per group). Magnification is 20×.

We next assessed whether the absence of IL-17A was associated with a reduction in inflammatory cell infiltration in the foot tissue of CHIKV-infected mice. Histological analysis of the feet of CHIKV-infected mice at 10 dpi showed reduced leukocyte infiltration of the synovial space and interarticular muscle (IAM) tissue in the feet of *il17a^−/−^* mice, compared to WT mice (Fig. 3A), and automated quantification of leukocyte infiltrates indicated that this difference was statistically significant (Fig. 3B).

### Reduced neutrophils in IL-17A-deficient CHIKV-infected mice.

**FIG 4:**
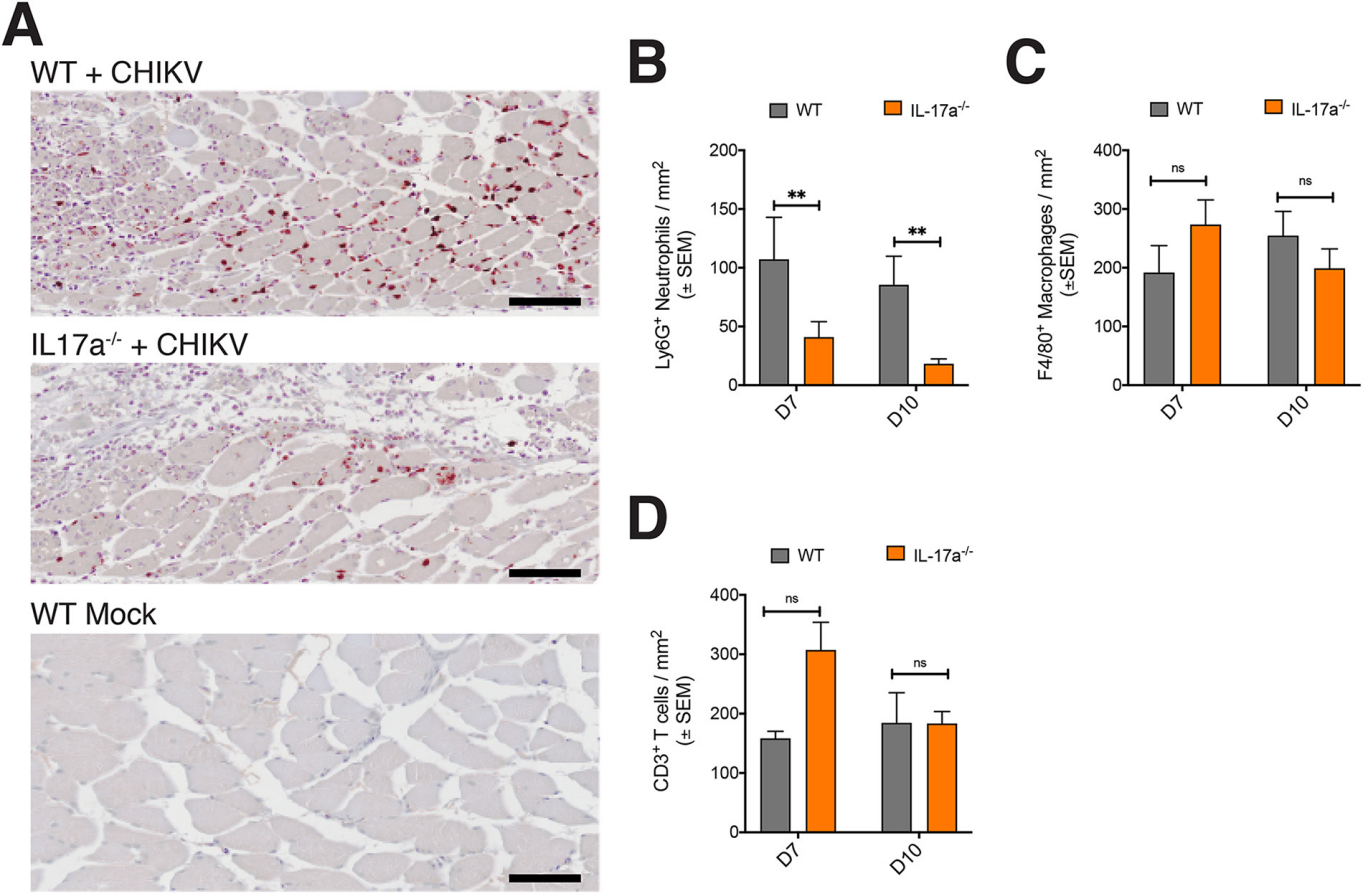
IHC of CHIKV-infected mouse feet. (A) Images of Ly6G staining of muscle from the feet of WT and *il17a^−/−^* mice on 7 dpi (+CHIKV), with an uninfected control (bottom). (B) Quantification of Ly6G-positive cells (neutrophils) per square millimeter of foot section (*n* = 7 *il17a^−/−^* mice and *n* = 4 C57BL/6J [WT] mice). **, Statistical analyses of differences between groups performed by independent-sample Mann-Whitney tests, *P = *0.023 for day 7 and *P = *0.018 for day 10. (C) Quantification of F4/80-positive cells (monocytes/macrophages) per square millimeter of foot section (*n* values as for panel B). Differences between groups were not significant (ns). (D) Quantification of CD3^+^ cells (T lymphocytes) per square millimeter of foot section (*n* values as for panel B). Differences between groups were not significant. Statistical significance between groups was assessed using a Mann-Whitney test, the scale bar represents 70 μm.

**FIG 5:**
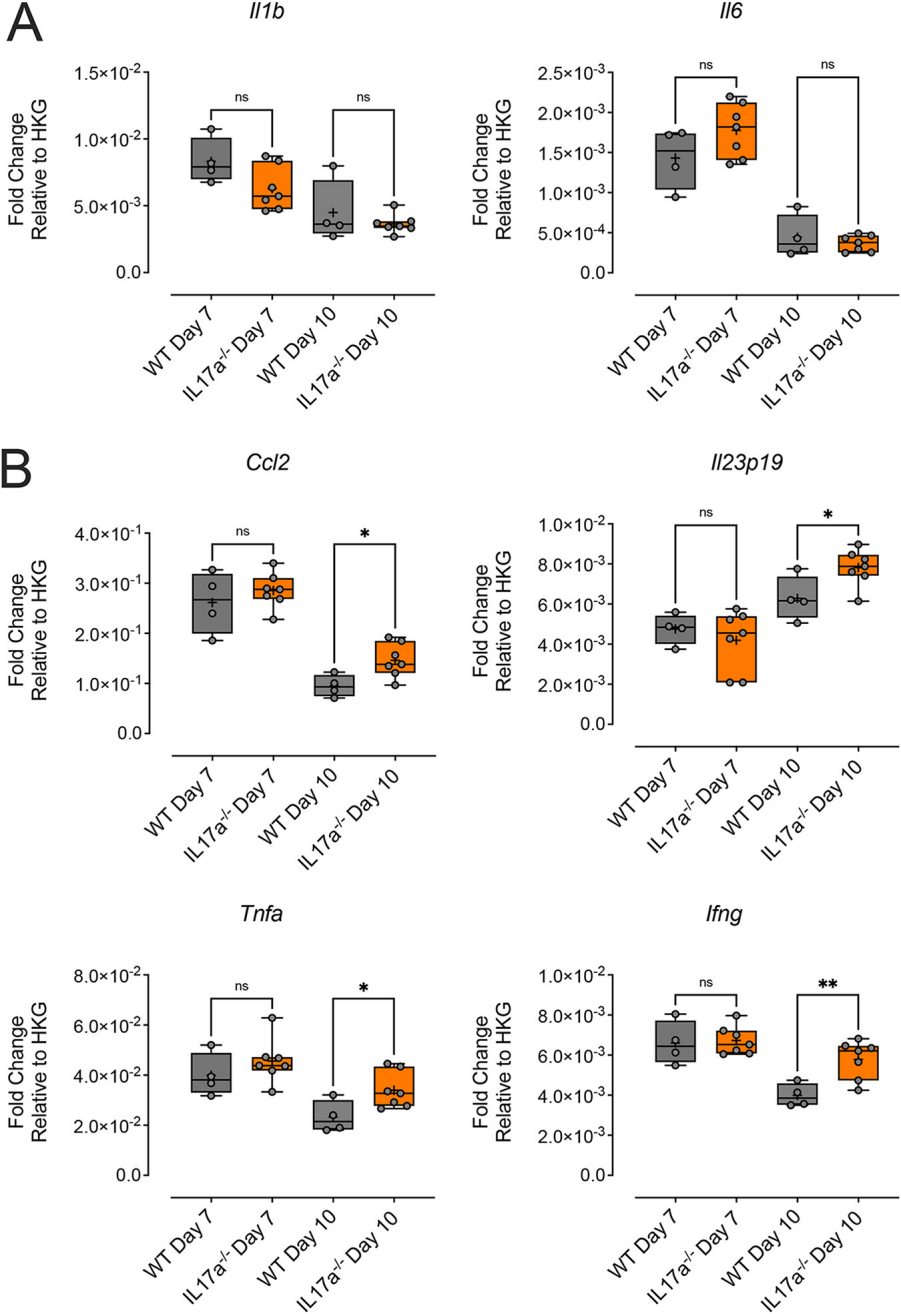
Relative mRNA expression of proinflammatory cytokines in the feet of WT and *Il17a^−/−^* mice. (A) Fold changes in mRNA expression (normalized to a housekeeping gene [HKG]) of *Il1b* and *Il6* in the feet of WT (gray boxes) and *Il17a^−/−^* (orange boxes) mice at 7 and 10 dpi. (B) Fold changes in mRNA expression of *Ccl2*, *Il23p19*, *Tnfa*, and *Ifng* in the feet of WT and *Il17a^−/−^* mice at 7 and 10 dpi. Values are shown as box-whiskers plots, with the central line showing the median and mean shown as a plus sign. mRNA levels were normalized to *Rpl13a* (housekeeping gene [HKG]). Statistically significant differences (*P < 0.05*) between groups were determined using an ordinary one-way ANOVA. ns, Non-statistically significant differences. *, *P* < 0.05; **, *P* < 0.01. Data shown are representative of two experiments (WT mice, *n* = 4 per time point; *il17a^−/−^* mice, *n* = 7 per time point).

To determine whether the observed differences in foot swelling were due to changes in the inflammatory arthritic infiltrates, foot sections from WT and *il17a^−/−^* mice day 7 and 10 dpi were analyzed by immunohistochemistry (IHC) for neutrophils, monocytes/macrophages, and T cells. Neutrophils were more abundant in the feet of WT mice, compared to *il17a^−/−^* mice (Fig. 4A), and quantification of Ly6G-labeled neutrophils showed a statistically significant reduction in neutrophil density in the feet of *il17a^−/−^* mice (Fig. 4B), compared to WT mice. There were no significant differences in the numbers of infiltrating F4/80^+^ macrophages (Fig. 4C) or CD3^+^ T-cell infiltration (Fig. 4D) in the feet of WT versus *il17a^−/−^* mice. Interestingly, mRNA expression of proinflammatory cytokines in the feet of *il17a^−/−^* mice did not substantially differ from that in WT mice; a marginal reduction in *Il1b* expression in the feet of *il17a^−/−^* mice was observed at both 7 and 10 dpi, but this was not statistically significant (7 dpi, *P* = 0.531) (Fig. 5A). In addition, while *il6* levels were modestly higher in the feet of *il17a^−/−^* mice at 7 dpi, compared to WT mice, these levels were similar by 10 dpi (Fig. 5A). Other proinflammatory cytokine genes, including *Ccl2*, *Il23p19*, *Tnfa*, and *Ifng*, were expressed at similar levels in *il17a^−/−^* and WT mice at 7 dpi (Fig. 5B). By 10 dpi, however, we observed statistically significant upregulation of these cytokines in the feet of *il17a^−/−^* mice (Fig. 5B).

## DISCUSSION

Experimental models of CHIKV and other arthritogenic alphavirus-induced diseases have provided insights into the inflammatory pathways driving alphaviral arthropathies, thereby identifying potential therapeutic targets (2). For instance, CD4^+^ T-cell activation and migration were found to be important in the development of CHIKV-induced inflammation (8, 9), and interfering with CD4^+^ T-cell egress led to reduced tissue inflammation (10). In this study, we show that IL-17A, a cytokine important in arthritic pathologies such as RA and psoriatic arthritis (PsA) (6), also has a role in alphavirus-induced musculoskeletal immunopathology. Alphavirus-induced arthritis shares several gene expression and immunopathological features with RA (9), and IL-17A is a key contributor to arthritic inflammation in RA and PsA. IL-17A can exacerbate tissue inflammation by upregulating proinflammatory cytokines such as TNF-α and IL-1β and can contribute to osteoclastogenesis and bone resorption by promoting the expression of RANKL (13), all of which are also important in alphavirus infection.

Serum IL-17A levels were significantly higher in individuals with chronic CHIKVD (average of 143 days postonset), compared to those with acute CHIKVD, suggesting that, if an IL-17-dependent response is initiated in the acute phase of inflammation, it could be sustained through the chronic phase and contribute to long-term illness. More importantly, the pattern by which cytokine levels were higher in chronic CHIKV patients was also observed with cytokines that are essential for the initiation (IL-6 and IL-23) and maintenance (IL-21, IL-22, and IL-23) of IL-17A-driven responses, and the concomitant elevation of all of these associated cytokines suggests that transcriptional regulators (such as signal transducer and activator of transcription 3 [STAT3] and retinoic acid-related orphan receptor γt [RORγt]) that govern the transcription of these cytokines are important in CHIKVD. These findings are in agreement with those for a patient cohort from Singapore, in which high levels of IL-17 were identified in the plasma of patients with chronic CHIKVD 2 to 3 months after illness onset (14). Of note, we observed that the mean age of the patients in the chronic CHIKVD cohort who exhibited inflammatory symptoms (arthralgia and fever) was higher than that of the patients in the acute CHIKVD group, and this is consistent with reports that age (in particular, age of >45 years) represents a risk factor for developing chronic CHIKVD (3).

IL-17A is produced by several immune cell subsets but is most commonly associated with T lymphocytes, particularly CD4^+^ T cells, which can differentiate into the IL-17-producing Th_17_ helper subset, and T_17_ (or Tc_17_) CD8^+^ T cells. Other subsets, such as γδ T cells, ILCs, and neutrophils, have been shown to produce IL-17 under certain physiological conditions (11, 15), and a range of studies have implicated IL-17-producing cells as effectors of pathology (6).

Using a mouse model of CHIKV-induced inflammation, tissue mRNA expression of genes associated with IL-17 signaling, such as *Il23r*, *Il6*, and *Il17f*, was elevated in the feet of CHIKV-infected mice. mRNA levels of *Ifng*, a potent T-cell cytokine, were also upregulated. This is likely due to the concurrent action of several T-cell-derived cytokines in a tissue microenvironment in which large leukocyte infiltrates (including T cells) drive inflammation and tissue damage. The absence of IL-17A was associated with reduced tissue swelling during acute disease, as seen previously (16). Foot swelling in infected *il17a^−/−^* mice was significantly reduced, compared to that in infected WT mice, toward the late phase of acute infection (postacute disease), reinforcing the notion of a partial but physiologically relevant contribution of IL-17 receptor signaling in acute CHIKV-induced arthritic inflammation. Given the role of IL-17 receptor signaling in postacute disease recovery in mice and the elevated serum IL-17A levels in patients with chronic CHIKVD, it will be important to comprehensively assess long-term immune responses and the persistence of CHIKV RNA in *il17a^−/−^* mice during CHIKV infection to understand the mechanisms underlying chronic CHIKVD.

The absence of IL-17A-mediated signaling did not affect viremia, although we did note impaired clearance of CHIKV RNA in the feet after the peak of arthritic foot swelling and after the resolution of viremia. While no infectious virus could be recovered from feet during postacute disease recovery (10 dpi), the higher levels of CHIKV RNA in the tissue may be a consequence of impaired immunity, such as CHIKV-specific T-cell responses, in the absence of IL-17A. In this regard, no differences in the numbers of infiltrating monocytes/macrophages or CD3^+^ T cells in the feet of WT versus *il17a^−/−^* mice were observed. In accordance with previous studies (16), significantly reduced numbers of neutrophils in the feet of infected *il17a^−/−^* mice, compared to infected WT mice, were observed. Acute inflammation dominated by neutrophils is typical of IL-17-driven inflammation (17), and neutrophils are known to play a critical role in CHIKV-induced inflammation and bone erosion (18, 19). We observed that, in the feet of *il17a^−/−^* mice, there were modest (not statistically significant) reductions of *Il1b* expression at both 7 and 10 dpi, compared to WT mice. This is in line with reports that neutrophils can express *Il1b* in several pathologies, including inflammation and tumorigenesis (20, 21). Importantly, the possibility that IL-17-expressing neutrophils play a role in this model is consistent with reports that IL-6, which was upregulated in both patients and mice infected with CHIKV, is a critical regulator of neutrophils during IL-17-driven inflammation. The reduction in neutrophils during postacute disease correlates with reduced swelling in the feet of infected *il17a^−/−^* mice at this time point. Our observation that *Il6* expression was moderately upregulated in the feet of *il17a^−/−^* mice at 7 dpi - but not at 10 dpi - points toward a possible dysregulation of the proinflammatory cytokine microenvironment in the absence of IL-17 expression.

Reducing inflammation without compromising the host’s ability to control viral replication is clearly key to the development of safe interventions, and our data show that IL-17 has little effect on viremia, with an effect only on the persistence of CHIKV RNA. Interestingly, we also observed significant upregulation of cytokines associated with the host antiviral responses in the feet of *il17a^−/−^* mice at 10 dpi (but not 7 dpi); upregulation of *Ccl2*, *Il23p19*, *Tnf*, and *Ifng* was concomitant with a greater viral load in *il17a^−/−^* mice at 10 dpi, which suggests that, in the absence of IL17A, a positive feedback mechanism aimed at enhancing clearance of persistent virus may account for elevated expression of these cytokines during the postacute phase of CHIKVD. While the present study is consistent with the established relationship between IL-17 signaling and neutrophil-mediated inflammation in noninfectious arthritides and CHIKV arthritis, a recent study by Neupane et al. highlights a role for IL-17A in inhibiting interferon alpha-2 (IFN-α2) expression during CHIKV infection (16), reinforcing the notion that IL-17 signaling most likely acts in cooperation with a number of transcriptionally unrelated pathways, including the type I IFN antiviral signaling pathway. It is worth noting that some differences between the findings in the study by Neupane et al. (16) and our study may provide some understanding of how IL-17 is implicated in CHIKVD; specifically, Neupane et al. (16) principally reported a clear reduction in foot swelling during the first peak (2 to 3 dpi) following CHIKV subcutaneous infection. This peak represents the first inoculation-induced edema of the biphasic response to CHIKV infection in mice (12), but whether the reduction in acute foot swelling at 7 dpi in *Il17a−/−* mice reported by Neupane et al. (16) is statistically significant is not clear. Although we similarly did not detect a significant reduction in foot swelling during the second peak (6 to 7 dpi) of CHIKVD in *il17a^−/−^* mice, we did observe a statistically significant reduction in foot swelling from 10 dpi to 12 dpi, whereas the study by Neupane et al. (16) did not. Interestingly, a more pronounced reduction in foot swelling observed in *Il17ra^−/−^* mice in the postacute phase may point toward a potential role for other isoforms of IL-17, such as IL17A, IL17F, and IL17A/F heterodimers (22), that engage the IL-17 receptor. Another important difference was the finding by Neupane et al. (16) that, at 1 dpi, the CHIKV RNA load in the serum was significantly reduced in *Il17a^−/−^* mice, compared to WT mice. In contrast, we assessed serum CHIKV RNA expression kinetics from 0 dpi to 6 dpi and did not observe any differences; one possible explanation for this difference may lie in the lower inoculation titer of CHIKV used in our experiment (10^4^ PFU), compared to that used by Neupane et al. (16) (10^5^ PFU), which might have overwhelmed antiviral responses in *Il17a^−/−^* mice at such an early time point.

More studies are needed to determine the role of IL-17 signaling in antiviral responses following arbovirus infection, particularly in the context of chronic CHIKVD. There are FDA-approved biologics targeting IL-17A, such as secukinumab, ixekizumab, bimekizumab, and brodalumab (5), raising the possibility that such drugs might find utility in the treatment of chronic CHIKV arthropathy. However, our findings that CHIKV RNA levels are increased in the tissues of *Il17a^−/−^* mice in the postacute phase indicate that, although there is no clear established link between late CHIKV RNA persistence and chronic CHIKVD, a high degree of caution should be applied in considering the use of drugs that target IL-17 to treat chronic CHIKV-induced arthritis.

## MATERIALS AND METHODS

### CHIKVD subjects.

Serum samples from CHIKV-infected patients were obtained from a cohort of 53 patients (including 13 healthy controls) who were diagnosed at the University Hospital in Aracaju, Sergipe State, Brazil. Patients were diagnosed with CHIKV infection based on clinical symptoms, fever, skin exanthem, serological findings, and positive qRT-PCR test results (see Table S1 in the supplemental material). The study was approved by the human ethics committee of the Federal University of Sergipe University Hospital, Brazil (reference number 1.486.302). All patients signed informed consent forms. The sera were obtained just after disease onset (acute) and during the chronic stage; the mean times between disease onset and sample collection were 2.2 days and 143.9 days for the acute and chronic cohorts, respectively. The average age of the cohort was 38.5 years, and the distribution was 77.3% female and 22.7% male. There was no significant difference in the ages of infected patients in relation to the control group; however, the ages of patients in the chronic phase of CHIKV infection were greater than the ages of patients in the acute phase of CHIKV infection (*P* = 0.02). There was a predominance of females in all groups, i.e., 78.79% of the infected cohort and 69.23% of the participants in the control group, with no statistically significant differences. Patients were monitored and treated for chronic arthritis with recommended drugs.

### Serum cytokine quantification.

Serum samples were collected from patients, and levels of soluble IL-17A, IL-6, IL-21, IL-22, and IL-23 were detected using a human ELISA kit (BD Biosciences). All ELISAs were performed according to the manufacturer’s instructions.

### Virus.

Stock of the La Reunion CHIKV isolate LR2006-OPY was grown, used, and titrated by 50% cell culture infectious dose (CCID_50_) assays using C6/36 cells and Vero cells, as described previously (8, 12).

### Plaque assay.

Vero E6 cells were seeded in 12-well plates at 2 × 10^5^ cells per well and cultured overnight at 37°C in Dulbecco’s modified Eagle’s medium (DMEM) with 5% fetal calf serum (FCS). The cells were infected with a dilution series of virus from samples and were overlaid with 1.2% colloidal microcrystalline cellulose (Sigma-Aldrich). The plates were incubated for another 72 h at 37°C. Cells were then fixed with 4% paraformaldehyde (PFA) and stained with 0.1% crystal violet. Viral titters were calculated by using the following formula: PFU/mL = (average number of plaques/volume [mL] of virus added) × dilution factor.

### Mice and viral infections.

WT C57BL/6J mice and IL-17A-deficient C57BL/6 mice (denoted *il17a^−/−^*) (23) were used in this study. All animal experiments were conducted in accordance with the Australian Code for the Care and Use of Animals for Scientific Purposes of the National Health and Medical Research Council of Australia. CHIKV work was approved by the QIMR Berghofer Medical Research Institute (QIMRB) animal ethics committee (reference number P2235) and were conducted in a biosafety level 3 (physical containment level 3 [PC3]) facility at QIMRB. For CHIKV infections, 6- to 8-week-old mice (with the same gender and age distribution in each group) were inoculated subcutaneously on the ventral side of the foot with 10^4^ PFU CHIKV diluted with PBS in a 50-μL volume, as described previously (12).

### mRNA expression analysis.

Ankle joints and quadriceps muscles were homogenized in TRIzol to extract total RNA. Total RNA was reverse transcribed into cDNA, and SYBR green-based qRT-PCR was performed using QuantiTect primers specific for *Il6*, *Il1b*, *Il23p19*, *Tnfa*, *Ifng*, *Il23r*, *Il17f*, and *Ccl2* (Qiagen) or forward and reverse primers (Sigma-Aldrich). Samples were normalized to a housekeeping gene (*Rpl13a*), and results were expressed as relative fold changes in expression relative to *Rpl13a* in mock-infected tissue using the 2^-ΔΔ^*^CT^* method (24). For the detection of CHIKV RNA in patients, serum samples were processed using a SuperScript III Platinum One-Step qRT-PCR system, and the following primers and probes were used: VCHIK NS4 6856F, TCACTCCCTGTTGGACTTGATAGA; VCHIK NS4 6981R, TTGACGAACAGAGTTAGGAACATACC; VCHIK NS4 6919p, 6-carboxyfluorescein (FAM)-AGGTACGCGCTTCAAGTTCGGCG. For the detection of CHIKV RNA in mice, feet were collected from mice and placed in RNAlater reagent prior to homogenization as described previously (9). CHIKV RNA was quantified by SYBR green qPCR using the following primers (5′ to 3′) targeting the CHIKV E1 gene: forward, AGCTCCGCGTCCTTTACC; reverse, CAAATTGTCCTGGTCTTCCTG.

### Histological staining and analysis.

Whole feet and quadriceps muscle tissues were fixed in 4% PFA and embedded in paraffin. Feet were decalcified in 14% EDTA prior to fixation. Five-micrometer-thick sagittal sections were stained with hematoxylin and eosin (H&E). Images were acquired using a BX53 Olympus microscope with a 10× objective and were analyzed using cellSens software. For automated quantification of nuclei, H&E-stained slides were scanned on an automated slide scanner (Aperio AT; Leica), images were analyzed using Aperio ImageScope software v12 (Leica), and nuclei were counted using the Nuclear v9 algorithm.

### IHC and image analysis.

Whole feet were examined by IHC as described previously (11). Sections were stained with rat anti-mouse Ly6G (catalog number NMP-R14; Abcam, Cambridge, MA, USA) (neutrophils), F4/80 (Abcam) (macrophages), or CD3 (Abcam) (T lymphocytes). Detection used NovaRED secondary antibody (ImmPACT NovaRED peroxidase substrate kit [catalog number SK-4805]; Vector Laboratories, Burlingame, CA, USA). Slides were digitally scanned using an Aperio AT Turbo system (Leica Biosystems), images were analyzed using Aperio ImageScope software (Leica), and cell quantitation was undertaken using open-source image analysis software, QuPath v0.2.3 (25). Three sections per foot were analyzed to produce a mean value for each mouse.

### Statistical analysis.

Statistical significance concerning the age distribution of the CHIKV-infected patient cohort was assessed by *t* test. Cytokine levels in acute CHIKV patients and healthy controls and in chronic CHIKV patients and healthy controls were assessed by one-way analysis of variance (ANOVA). For animal studies, a *t* test was used if the difference in variances was smaller than 4, the skewness was greater than −2, and the kurtosis was smaller than 2. Data assessed as nonparametric for which distributions were similar were subjected to a Kruskal-Wallis test; otherwise, a Kolmogorov-Smirnoff test was used. For mRNA expression values, statistical significance was assessed by a Mann-Whitney *U* test. For longitudinal foot swelling measurements, statistical significance between groups was assessed using a Kolmogorov-Smirnoff test. For the quantification of cells by IHC, the statistical significance of differences between groups was assessed by a Mann-Whitney *U* test. Statistical analyses were performed using SPSS or Prism v9.0 (GraphPad).

### Data availability.

All relevant data are provided within the paper and its supplemental material.
